# Effect of outdoor air pollution on asthma exacerbations in children and adults: Systematic review and multilevel meta-analysis

**DOI:** 10.1371/journal.pone.0174050

**Published:** 2017-03-20

**Authors:** Pablo Orellano, Nancy Quaranta, Julieta Reynoso, Brenda Balbi, Julia Vasquez

**Affiliations:** 1 Consejo Nacional de Investigaciones Científicas y Técnicas (CONICET), Buenos Aires, Argentina; 2 Universidad Tecnológica Nacional, Facultad Regional San Nicolás, San Nicolás, Argentina; 3 Comisión de Investigaciones Científicas (CIC), La Plata, Argentina; 4 Hospital Interzonal General de Agudos “San Felipe”, San Nicolás, Argentina; The Ohio State University, UNITED STATES

## Abstract

**Background:**

Several observational studies have suggested that outdoor air pollution may induce or aggravate asthma. However, epidemiological results are inconclusive due to the presence of numerous moderators which influence this association. The goal of this study was to assess the relationship between outdoor air pollutants and moderate or severe asthma exacerbations in children and adults through a systematic review and multilevel meta-analysis.

**Material and methods:**

We searched studies published in English on PubMed, Scopus, and Google Scholar between January 2000 and October 2016. Studies following a case-crossover design with records of emergency departments and/or hospital admissions as a surrogate of moderate or severe asthma exacerbations were selected. A multilevel meta-analysis was employed, taking into account the potential clustering effects within studies examining more than one lag. Odds ratios (ORs) and 95% confidence intervals were estimated. A subgroup analysis in children aged 0 to 18 years and a sensitivity analysis based on the quality of the included studies as defined in the Newcastle-Ottawa Scale were performed. Publication bias was evaluated through visual inspection of funnel plots and by a complementary search of grey literature. (Prospero Registration number CRD42015032323).

**Results:**

Database searches retrieved 208 records, and finally 22 studies were selected for quantitative analysis. All pollutants except SO_2_ and PM_10_ showed a significant association with asthma exacerbations (NO_2_: 1.024; 95% CI: 1.005,1.043, SO_2_: 1.039; 95% CI: 0.988,1.094), PM_10_: 1.024; 95% CI: 0.995,1.053, PM_2.5_: 1.028; 95% CI: 1.009,1.047, CO: 1.045; 95% CI: 1.005,1.086, O_3_: 1.032; 95% CI: 1.005,1.060. In children, the association was significant for NO_2_, SO_2_ and PM_2.5_.

**Conclusion:**

This meta-analysis provides evidence of the association between selected air pollutants and asthma exacerbations for different lags.

## Introduction

Asthma can be defined as a chronic inflammatory disorder of the airways associated with bronchial hyper-responsiveness, reversible airflow limitation and recurrent symptoms of wheezing, chest tightness, and cough [1]. Worldwide, asthma accounts for nearly 1% of all disability adjusted life years (DALYs) lost [2]. According to recent estimates, as many as 623 million people are currently living with some level of asthma-related symptoms [3], while 250,000 deaths can be attributed to this disease each year [4]. Economic losses due to asthma are estimated to be the highest among patients with chronic diseases due to significant healthcare utilization. Hospitalization and medications are the most important associated direct costs, while work and school absenteeism account for the greatest percentage of indirect costs [5]. On the other hand, asthma exacerbations are common in asthmatic patients [6], and the main goal of asthma treatment is the prevention of exacerbations and fixed airflow limitation [7].

Asthma exacerbations can be classified as mild, moderate, and severe, with the latter two generally requiring an emergency department visit and likely hospitalization [8]. In children, several risk factors for asthma exacerbations have been identified, including poor asthma control, individual susceptibility, viral infections, allergen exposure, environmental tobacco smoke (ETS) exposure, and outdoor air pollution [9].

Several studies have confirmed that air pollution from ozone (O_3_), sulfur dioxide (SO_2_), nitrogen dioxide (NO_2_), and particulate matter (PM) may induce or aggravate asthma [10]. A large number of observational studies have been conducted to assess the effect of air pollutants in asthma prevalence, incidence, and exacerbations. According to a number of published meta-analysis [11,12,13,14], these pollutants in the atmosphere are associated with higher incidence, prevalence, hospitalizations, or worsening of symptoms of asthma. Regarding the short-term effects of air pollutants in terms of exacerbations or worsening of symptoms, three studies have found an association with PM, NO_2_, SO_2_, carbon monoxide (CO) and O_3_ [15,16,17]. In one of these studies analyzing data from Asia, the association was not significant in people aged 15–64 years [17].

The present study provides complementary results to these previous meta-analyses, with two main methodological differences. First, our study has estimated a pooled association measure considering all lag times between air pollution increase and asthma exacerbations for each individual study, using a multilevel analysis. In contrast, previous meta-analyses either used only one lag for study, selecting them through predetermined rules of choice, or made subgroup analysis of specific lags. Second, our meta-analysis selected only studies following case-crossover designs, while previous meta-analyses included all time-series designs. The pros and cons of these choices are examined in the discussion section. Thus, the goal of this study was to assess the association between the increase in concentration of outdoor air pollutants and moderate or severe asthma exacerbations in children and adults, through a systematic review, multilevel meta-analysis, and meta-regression of case-crossover studies, pooling all the lag times and taking into account potential moderators.

## Materials and methods

We prepared this article according to the PRISMA guidelines for systematic reviews and meta-analysis [18] (S1 Table). The protocol for this study was registered in PROSPERO (http://www.crd.york.ac.uk/PROSPERO/) under registration number CRD42015032323 before the formal screening of search results (S1 File). A pilot of the study was previously carried out to adjust the search strategy.

### Search strategy and sources

We searched studies published in English in PubMed, Scopus, and Google Scholar between January 2000 and October 2016. The searched studies should explore the relationship between outdoor air pollution and acute exacerbations of asthma in children and adults through a case-crossover observational design. Moderate and severe exacerbations were represented as visits to emergency departments or hospitalizations by this cause. A combination of the following terms combined using Boolean connectors was used: “asthma”, “wheeze”, “pollut*”, “contamin*”, “hospitaliz*”, “admission*”, “emergenc*”, “attack*”, “case crossover”. A detailed description of the search strategy in PubMed, Scopus, and Google Scholar is shown in the supplementary material (S2 Table). In addition, we have manually searched in reference lists from other systematic reviews in order to find additional studies.

### Study selection

Two reviewers (PO and JR) independently screened all records retrieved from database searches in three stages. First they searched the assessment of titles, second the assessment of abstracts, and finally they screened the assessment of full-text articles. Any disagreement was resolved by consensus with the help of a third reviewer (BB). The inclusion criteria for the retrieved articles were: (1) studies following a case-crossover design; (2) assessment of outdoor air pollution represented by NO_2_, SO_2_, PM_10_, PM_2.5_, CO and O_3_; (3) studies working with records of emergency departments or hospital admissions as a surrogate of moderate or severe asthma exacerbations; (4) studies reporting odds ratio (OR) and 95% confidence intervals (95%CI) as the measure of association; and (5) articles published as a full-text document.

### Data extraction

Data were extracted by means of a data extraction form developed in Microsoft Excel®. These data included: (1) first author and year of publication; (2) participant’s age and gender; (3) OR and 95% confidence intervals (95%CI); (4) the lags, understood as the time distance between the increase of the selected pollutant and the date of the asthma exacerbation. Other variables considered were latitude and elevation of the study sites. These variables were used in the meta-regression analysis. In the case of multicity studies, a separate OR was extracted for each city. When this information was not available, the data was considered as missing.

### Risk of bias and quality assessment

Two reviewers (PO and JR) independently evaluated the methodological quality of each selected study, in order to determine the risk of bias. The Newcastle–Ottawa scale (NOS) for case-control studies was used as a measure of the quality of individual studies [19]. In this scale, there are three dimensions: selection of the study group, comparability of the groups, and exposure ascertainment. A total of seven questions are raised, with a minimum of zero and a maximum of nine stars. Study quality is then graded as poor (1–3 stars), intermediate (4–6 stars), or high (7–9 stars). An explanation of each question adapted for this study is presented in the supporting material (S3 Table). The potential risk of publication bias was evaluated by visual examination of funnel plots asymmetry [20]. In addition, a grey literature search was performed in order to identify studies from conference proceedings and other sources that may have had null results and were not published as articles in journals. The sources for the grey literature search were Google Scholar, Scopus, Biomed Central (http://www.biomedcentral.com) and NLM Gateway (https://gateway.nlm.nih.gov/gw/Cmd).

### Sensitivity analysis

We performed a sensitivity analysis based on the quality of the included studies as defined in the NOS. Studies classified with 6 or more stars (intermediate-high quality) were selected for the sensitivity analysis.

### Statistical analysis

The ORs and 95%CIs derived from single-pollutant models were retrieved and weighted based on the inverse of the variance method, assuming that observations with lower variances should be given more weight in the analysis. Where necessary, the coefficient estimates were recalculated to reflect a 10-μg/m^3^ increase in PM_10_ and PM_2.5_, a 10 ppb increase in NO_2_, SO_2_, and O_3_, and a 1 ppm increase in CO, assuming a linear relationship within the considered range. The majority of studies contributed with more than one lag between the increases in pollutant concentration up to the date of the asthma exacerbation. Accordingly, we used two or more effect sizes from each study, one for each lag. Thus, a multilevel meta-analytic model was employed, taking into account the potential clustering effects within studies examining more than one lag at a time. Other meta-analyses considered only one lag per study using a preset rule. On the contrary, the multilevel meta-analysis allows for the hierarchical structure of the data, in which lags are nested within studies. The multilevel model was then extended using a multilevel meta-regression model to test the modifying effect of the lag and other moderators, i.e. the latitude and elevation. The heterogeneity was measured with the Q_E_-test for residual heterogeneity, which tests whether the variability in the observed effect sizes that is not accounted for by the moderators is larger than one would expect based on sampling variability [21]. A subgroup analysis in children aged 0 to 18 years was also performed, to evaluate the possible association in this age group. All analyses were performed using the “metafor” package (version 1.9–4) [21] in the statistical software R, version 3.2.2 (https://www.r-project.org/) [22]. The latitude and elevation of the cities were obtained through packages “ggmap” [23] and “weatherr” [24] from the same software.

## Results

### Characteristics and quality of studies

**Fig 1:**
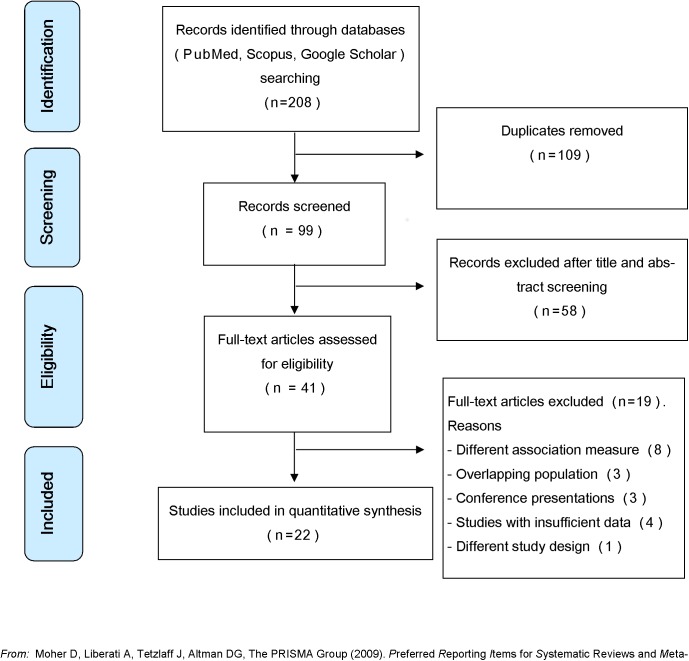
PRISMA flowchart of the study identification and selection process.

Database searches retrieved 208 records. After removing duplicates, title and abstract screen on 99 records were completed by reviewers, and 41 articles were selected for full-text eligibility assessment. Nineteen studies were excluded due to different reasons (Fig 1). Finally, 22 studies were selected for quantitative analysis (Fig 1), representing 267,413 visits to emergency departments, emergency calls, or hospitalizations due to asthma exacerbations in all age groups.

Of these studies, 12 analyzed only children and one analyzed only adults. The outcomes were hospitalizations (seven), emergency department visits (10), both (four) or emergency telephone calls (one). Studies were carried out in 12 countries. 10 were classified as high-income countries, and two were classified as upper-middle-income countries according to the World Bank classification [25]. The elevation of the cities where the studies were carried out were between eight and 1,134 meters over the sea level, and absolute latitudes were between 25° and 54°, while tropical climate zones were not represented. A general description of each included study can be seen in Table 1.

**Table 1:** Characteristics of the included studies.

First author, year	Country	Country classification	N	Ages (years)	Pollutants	NOS scale	Ref.
Alman, 2016	USA	High-income	1,136	0–≥ 65	PM_2.5_	5	[26]
Canova, 2012	UK	High-income	234	18–≥ 75	PM_10_	7	[27]
Chen, 2013	Taiwan	High-income	1,912	5–15	PM_2.5_, O_3_	6	[28]
Ding, 2016	China	Upper-middle-income	2,507	0–18	NO_2_, SO_2_, PM_10_, PM_2.5_, CO, O_3_	5	[29]
Glad, 2012	USA	High-income	6,979	0–≥ 75	PM_2.5_	6	[30]
Gleason, 2014	USA	High-income	21,854	3–17	PM_2.5_, O_3_	6	[31]
Grineski, 2011	USA	High-income	3,504	1–≥ 65	NO_2_, PM_2.5_	6	[32]
Iskandar, 2012	Denmark	High-income	8,226	0–18	NO_2_, PM_10_, PM_2.5_	5	[33]
Laurent, 2008	France	High-income	4,677	0–≥ 65	NO_2_, SO_2_, PM_10_, O_3_	5	[34]
Lavigne, 2012	Canada	High-income	3,728	2–≥ 60	NO_2_, SO_2_, PM_2.5_, CO, O_3_	6	[35]
Lewin, 2013	Canada	High-income	429	0–4	SO_2_, PM_2.5_	5	[36]
Li, 2011	USA	High-income	7,063	2–18	NO_2_, SO_2_, PM_2.5_, CO	6	[37]
Lin, 2003	Canada	High-income	7,319	6–12	NO_2_, SO_2_, CO, O_3_	5	[38]
Pereira, 2010	Australia	High-income	603	0–19	NO_2_, CO	5	[39]
Sacks, 2014	USA	High-income	121,621	0–≥ 65	O_3_	6	[40]
Santus, 2012	Italy	High-income	3,579	0–≥ 75	NO_2_, SO_2_, PM_10_, PM_2.5_, CO, O_3_	5	[41]
Smargiassi, 2009	Canada	High-income	1,842	2–4	SO_2_	6	[42]
Sunyer, 2002	Spain	High-income	4,635	14–≥ 80	NO_2_, SO_2_, PM_10_, CO, O_3_	8	[43]
Tecer, 2008	Turkey	Upper-middle-income	2,779	0–14	PM_10_, PM_2.5_	5	[44]
Ueda 2010	Japan	High-income	3,427	0 – 12	NO_2_, SO_2_, PM_10_	5	[45]
Villeneuve 2007	Canada	High-income	57,912	2–≥ 75	NO_2_, SO_2_, PM_10_, PM_2.5_, O_3_	6	[46]
Yamazaki 2015	Japan	High-income	1,447	0–14	NO_2_, PM_10_, PM_2.5_, O_3_	5	[47]

NO_2_: nitrogen dioxide, SO_2_: sulfur dioxide, O_3_: ozone, CO: carbon monoxide, PM_10_: particulate matter < 10 μm, PM_2.5_: particulate matter < 2.5 μm, N: number of emergency department visits, hospitalizations or participants, NOS scale: Newcastle-Ottawa scale.

The lags considered included single-day lags (0 to 6 days) and cumulative lags (1 to 6 days moving average) before the date of the event. The majority of studies assessed the effect of more than one pollutant (16 studies), while eight studies considered two or multiple pollutant models in addition to single pollutant models. All studies employed generalized linear model (GLM) techniques to estimate the regressions, but two studies also used a generalized additive model (GAM). The other variables considered for adjusting the regressions were temperature, relative humidity, dew point, barometric pressure, wind speed, global radiation, cloudiness, and one study took into account the influenza and soybean asthma outbreaks, while four studies failed to consider the adjustment for other variables.

According to the NOS, 20 studies were classified as intermediate quality, and the remaining two studies were classified as high quality. The two main problems related to studies’ quality were the adequate case definition and the ascertainment of exposure, because in most articles there was no independent validation and the residential address of cases was not verified.

### Data preprocessing, heterogeneity, and publication bias

In quantitative analysis, the 22 studies contributed with 345 effect sizes corresponding to NO_2_ (68), SO_2_ (60), PM_10_ (44), PM_2.5_ (68), CO (50) and O_3_ (55) were included.

Based on the results of the meta-regression, the lag significantly affected the relationship between the increases in pollutant concentrations and severe asthma exacerbations for the NO_2_ and the O_3_ (Table 2). For SO_2_, PM_10_, PM_2.5_ and CO, the lag had no influence on the associations. Other moderators that influenced these associations were the elevation over the sea level for the PM_10_ and the latitude for the PM_10_, PM_2.5_ and CO (Table 2). The Q_E_ for each pollutant showed a significant heterogeneity for the SO_2_, PM_10_, PM_2.5_, and O_3_, which could possibly indicate that other moderators not considered in the models are influencing the association between these pollutants and the occurrence of asthma exacerbations (Table 2).

**Table 2:** Multilevel meta-regression analysis.

Pollutant	Q_E_ (P-value)	Moderator	P-value
NO_2_	0.33	Lag	<0.01
		Latitude	0.80
		Elevation	0.14
SO_2_	0.02	Lag	0.36
		Latitude	0.51
		Elevation	0.16
PM_10_	<0.01	Lag	0.4
		Latitude	<0.01
		Elevation	<0.01
PM_2.5_	<0.01	Lag	0.76
		Latitude	0.01
		Elevation	0.81
CO	0.85	Lag	0.39
		Latitude	0.01
		Elevation	0.55
O_3_	0.03	Lag	<0.01
		Latitude	0.22
		Elevation	0.54

NO_2_: nitrogen dioxide; SO_2_: sulfur dioxide; O_3_: ozone; CO: carbon monoxide; PM_10_: particulate matter < 10 μm; PM_2.5_: particulate matter < 2.5 μm; Q_E_: test for residual heterogeneity.

**Fig 2:**
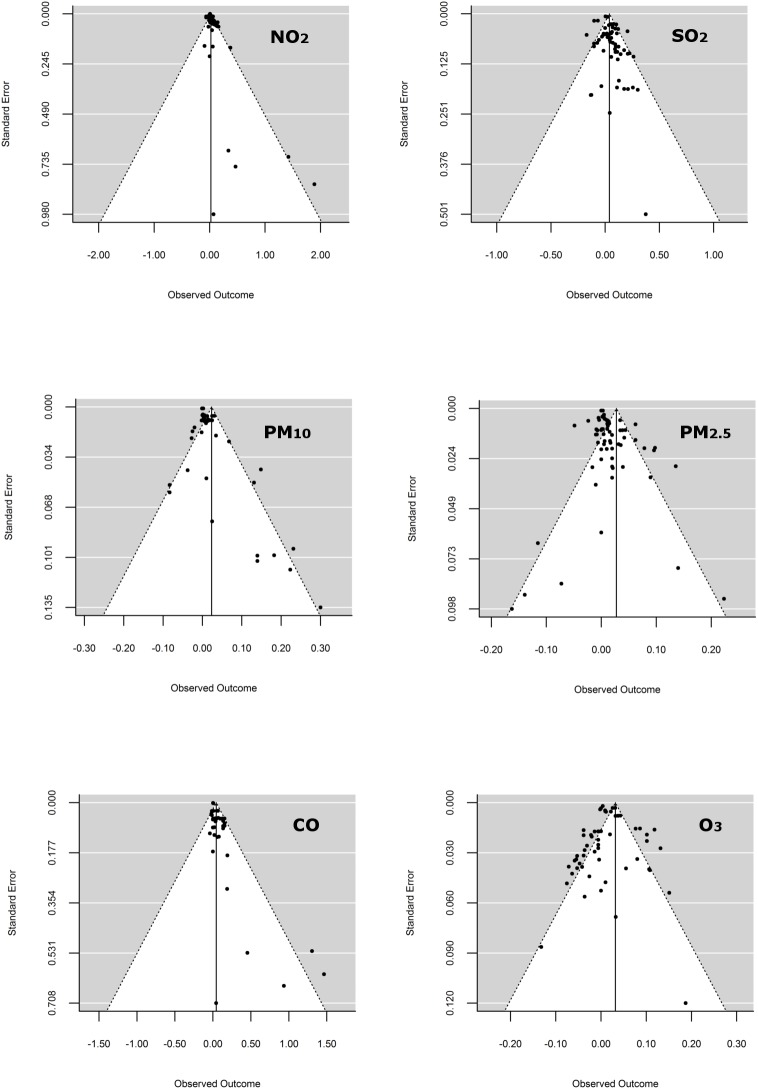
Funnel plot to explore publication bias for each pollutant. The figure shows the observed outcomes (Log odds ratios) on the horizontal axis against their corresponding standard errors.

An asymmetry in data points can be observed for NO_2_, PM_10_, and CO, where the absence of negative outcomes produced by less precise studies suggested potential publication biases (Fig 2).

The plots show the observed outcomes (Log odds ratios) on the horizontal axis against their corresponding standard errors for nitrogen dioxide (NO_2_), sulfur dioxide (SO_2_), ozone (O_3_), carbon monoxide (CO), particulate matter < 10 μm (PM_10_) and particulate matter < 2.5 μm (PM_2.5_). The search of grey literature identified seven additional studies not included in our analysis; five were conference abstracts [48–52] and two were articles published in local languages (Italian and Korean) [53,54]. In none of these studies the association of pollutants with asthma exacerbations was negative, null or non-significant.

### Association between pollutants and asthma exacerbations

The only pollutants that did not show a significant association with asthma exacerbations within the considered lags were SO_2_ (OR: 1.039; 95% CI: 0.988,1.094) and PM_10_ (OR: 1.024; 95% CI: 0.995,1.053), while this association was significant for NO_2_ (OR: 1.024; 95% CI: 1.005,1.043), PM_2.5_ (OR: 1.028; 95% CI: 1.009,1.047), CO (OR: 1.045; 95% CI: 1.005,1.086), and O_3_ (OR: 1.032; 95% CI: 1.005,1.060). Figures in S2 File show forest plots with adjusted ORs and their 95%CI for the associations between pollutants and asthma exacerbations, as reported in each original article. In the subgroup analysis of children aged 0 to 18 years, the association was significant for NO_2_ (OR: 1.040; 95% CI: 1.001,1.081), SO_2_ (OR: 1.047; 95% CI: 1.009,1.086), and PM_2.5_ (OR: 1.022; 95% CI: 1.000,1.045). A subgroup analysis of adults was not performed as only one study was focused exclusively on this age group, while few studies reported results of adults separately from those of children.

### Sensitivity analysis

For the sensitivity analysis, 11 studies with a NOS scale ≥ 6 (intermediate-high quality) were selected, representing 113 effect sizes. According to these analyses, the association was significant for NO_2_ (OR: 1.009; 95% CI: 1.004,1.013), PM_2.5_ (OR: 1.023; 95% CI: 1.006,1.041) and O_3_ (OR: 1.020; 95% CI: 1.013,1.027). Heterogeneity could be controlled after the sensitivity analysis for all pollutants (S4 Table). Publication bias in this subanalysis was not evaluated due to the more reduced sample size.

## Discussion

This meta-analysis showed a significant association between the main pollutants, with the exception of SO_2_ and PM_10_, and moderate or severe exacerbations of asthma. Moreover, this approach allowed the simultaneous analysis of all lags considered in different studies, obtaining one polled measure of association. In the subgroup analysis of children, the exacerbations were associated with NO_2_, SO_2_ and PM_2.5_. In the sensitivity analysis, where only studies with a NOS scale ≥ 6 were selected, the association was significant for NO_2_, PM_2.5_ and O_3_. The most important outdoor air pollutants are PM, O_3_, SO_2_, NO_2_, CO and Lead (Pb) [55]. The main anthropogenic sources of PM are traffic and transportation, electricity generation and other combustion processes [56]. With respect to gases, the main sources of SO_2_ in the developed world are primary emissions during energy production or industrial processes [57], while NO_2_ and CO are principally emitted from fossil fuel combustion in urban environments [58]. Ozone is a secondary pollutant formed by photochemical reactions between sunlight and pollutant precursors, such as nitrogen oxides and volatile organic compounds [59]. Increased pollution exposures have been associated with increased numbers of hospital admissions and emergency-room visits, mainly due to exacerbations of chronic obstructive pulmonary disease (COPD) and asthma [60]. Air pollution may be related to asthma exacerbations through oxidative stress, airway remodeling and inflammation, and sensitization to aeroallergens [61,62]. Further, pulmonary inflammation can indirectly influence the worsening of asthma symptoms by affecting host defenses [63] and enhancing infections with rhinovirus (RV), influenza, and respiratory syncytial virus (RSV), which in turn are considered the main cause of asthma exacerbations [64]. Specifically, O_3_ exposure causes airway inflammation, airway hyper-responsiveness, and decrements in lung function, while SO_2_ mainly leads to bronchoconstriction [64] and NO_2_ probably triggers bronchial inflammation as a precursor of O_3_ [64]. On the other hand, exposure to PM might cause oxidative stress, airway hyper-responsiveness, and airway remodeling, either alone or in combination with allergic sensitization [65]. In the atmosphere, different PM sizes can be found. The coarse fraction (PM_10–2.5_) can penetrate into the upper airways, but the fine fraction (PM_2.5-1_) can be deposited in the lung, especially in the alveoli, although it could pass to the systemic circulation [63]. It should be noted that besides the size of PM, the chemical composition is very important to understand the health effects of particulate matter. There are also differences in the individual susceptibility to air pollutants. Children are more affected than adults and boys more affected than girls, while a diet high in fruits and vegetables and of antioxidant vitamin supplements could be a protective factor, and obesity might increase susceptibility to the adverse effects of air pollution [62].

In this article, the heterogeneity of the included studies was significant for SO_2_, PM_10_, PM_2.5_ and O_3_, and could not be completely controlled by the modifying effect of the moderators that were considered here, including the lag, the elevation and the latitude of the cities that were under analysis. It is possible that other effect modifiers, which were not available in the included articles, could have influenced these results. However, the heterogeneity was resolved for all pollutants in the sensitivity analysis.

The funnel plots showed a degree of asymmetry for the NO_2_, the PM_10_ and the CO. This asymmetry may suggest that small studies showing no statistically significant effects remain unpublished, and then the true effect could be overestimated [66]. Another possible explanation could be that small studies may have weaker methodological quality than larger studies, leading to an overestimation of the true effects [67]. Regarding the first possibility, our complementary search of grey literature allowed us to identify seven additional studies with the same design and objectives. However, in these studies the association between air pollutants and asthma exacerbations was positive and significant, meaning that the reason to be unpublished in journals was not related to null results. As to the second possible explanation, for NO_2_ and CO all the effect sizes that presented the highest standard errors came from a single study [39] that showed a low value (5 stars) of the NOS scale. Nonetheless, for PM_10_ the effect sizes that showed the maximum values of standard errors came from one study that was classified as high-quality [27] according to the NOS scale. It is worth noting that this study was the one that included only adults. Taking these results into consideration, it is possible that the asymmetry of funnel plots showed by the NO_2_ and the CO can be due to a weak methodological quality of one study which contributes with 5 observations, and not to publication bias. On the other hand, for PM_10_ the funnel plot asymmetry does not seem to be related to methodological quality, and thus publication bias cannot be ruled out.

In general, the relatively small risks and ORs detected through this meta-analysis may lead one to assume that the potential effect of outdoor air pollutants at a population level is negligible, and thus the impact of public health measures could be dismissed. However, nearly 300 million people suffer from asthma globally [4]. A large amount of people are susceptible to moderate and severe exacerbations related to outdoor air pollution, and a very large number of people are exposed to outdoor pollutants, for example by living near polluted roads. Considering these two facts, a combination of small relative risks and high prevalence of exposure can contribute to a moderate population attributable fraction. Thus, a public health intervention aimed at mitigating the effects of air pollutants and targeted to the entire population might have significant benefits for the society.

This study had several limitations. The first was the use of non-randomized observational studies that failed to control bias due to the confounding effects of several factors. The second limitation was that the majority of studies which were included used secondary data sources for the asthma hospitalizations and emergency visits. These two points do not allow the control of confounding factors, for example by controlling the change in medications or the exposure to secondhand smoke in the evaluated cases. A third limitation was that air pollutant concentrations were estimated in a different way in different studies, such as using alternative models or an unequal number of monitoring sites. Besides, the lags considered were often different, and these points would have caused the high heterogeneity between studies. The limited number of moderators could be considered the fourth limitation, as there are probably several other factors that modify the association between air pollutants and asthma exacerbations. One of these factors is the country income: almost all studies were conducted in high-income countries, and accordingly the effect of these pollutants in the asthma exacerbations could not be extrapolated to developing countries. Finally, this article only considered studies following a case-crossover design. This might be seen as a limitation in the sense that a number of studies have likely been excluded from the analysis, particularly time-series studies. The equivalence of results obtained from time-series Poisson regression and from case-crossover studies using conditional logistic regression has been proposed by Lu et al. [68]. However, there exist at least three differences between these methods. First, case-crossover designs appear to be less efficient than Poisson time-series designs, i.e. showing lower statistical power [69–71]. Second, case-crossover designs are less vulnerable to bias [69–71] and, unlike time-series analyses, individual data can be included to estimate effect modifications and to control for confounders by design [72]. Third, time-series regression based on generalized additive models involves several arbitrary decisions, e.g. the type of smoother or the number of degrees of freedom [69]. Carracedo-Martínez et al. [72] performed a thorough review of the literature comparing these methodologies. Taking these points into consideration, both study designs are potentially exposed to different biases, are subject to other assumptions, and display a dissimilar efficiency. Accordingly, we consider that our choice of a unique study design could be considered as a major strength, because it enables the obtention of more compatible and precise association values. However, our results should be interpreted as complementary and not antagonistic to other broader studies that included both case-crossover and time-series designs.

## Conclusions

In conclusion, this meta-analysis provides evidence of the association between major air pollutants and moderate or severe asthma exacerbations. Moreover, this study proposes a methodological approach to obtain a single association value pooling different lags and studies, through the use of a multilevel meta-analytic model. Other similar studies should be carried out to confirm or discard the present findings, to update these results, and to achieve more accurate association values. This article also highlights the importance of confounders in the association of air pollutants and asthma exacerbations. The implications of these results for public health interventions and individual prevention are also suggested. Finally, it was pointed out that almost all observational studies included in this meta-analysis come from developed countries, and therefore extrapolating these results to developing countries requires caution. In this sense, the development of similar studies should be promoted to evaluate the associations between air pollutants and asthma under different income scenarios.

## Supporting information

S1 File PROSPERO protocol. (PDF)

S2 File Forest plots of selected pollutants. (PDF)

S1 Table PRISMA Checklist. (PDF)

S2 Table Detailed literature search. (PDF)

S3 Table Newcastle-Ottawa criteria. (PDF)

S4 Table Heterogeneity test for moderators (lags, latitude, elevation) according to the multilevel meta-regression model in the sensitivity analysis. (PDF)
