# Supplementary material for: Effect of outdoor air pollution on asthma exacerbations in children and adults: Systematic review and multilevel meta-analysis
Source: PLoS One. 2017 Mar 20;12(3):e0174050. doi: 10.1371/journal.pone.0174050 (PMC5358780; doi:10.1371/journal.pone.0174050)
Supplement: S1 File — (PDF) [file pone.0174050.s001.pdf]

PROSPERO International prospective register of systematic reviews

Effect of outdoor air pollution on severe asthma exacerbations: systematic review and meta-analysis of case-crossover studies

Pablo Orellano, Julieta Reynoso, Brenda Balbi, Julia Vasquez, Nancy Quaranta

Citation

Pablo Orellano, Julieta Reynoso, Brenda Balbi, Julia Vasquez, Nancy Quaranta. Effect of outdoor air pollution on severe asthma exacerbations: systematic review and meta-analysis of case-crossover studies. PROSPERO 2015:CRD42015032323 Available from
http://www.crd.york.ac.uk/PROSPERO_REBRANDING/display_record.asp?ID=CRD42015032323

Review question(s)

What is the magnitude of the short-term effects of outdoor air pollutants (NO2, SO2, CO, O3, PM10 and PM2.5) on severe asthma exacerbations?

Searches

A search for published research articles will be conducted in the following bibliographic databases: MEDLINE via PubMed, Scopus, and Google Scholar. The search strategy will include terms related to the disease, the exposure factors, and the design of the studies.

Studies published between January 2000 and the date of the search will be retrieved and independently assessed for eligibility by two reviewers. Only articles published in English will be considered.

An example of the search strategy is presented below.

Pubmed: asthma[All Fields] AND "case crossover"[All Fields] AND (pollut*[All Fields] OR contam*[All Fields]) AND (hospitali*[All Fields] OR admissio*[All Fields] OR emergency[All Fields] OR attac*[All Fields]).

Google Scholar: "case crossover" AND intitle:asthma AND intitle:pollution AND (intitle:hospital OR intitle:hospitalizations OR intitle:admissions OR intitle:emergency OR intitle:attack).

Types of study to be included

In order to increase the precision of the effect sizes, only studies of the same design will be included. Therefore, exclusively case-crossover studies reporting odds ratio (OR) as the measure of association will be considered, preferably with bi-directional selection of control periods.

Condition or domain being studied

Severe asthma exacerbations. An asthma exacerbation is defined as an episode of wheezing or shortness of breath in a person with asthma, and may include the need for oral or systemic corticosteroids. A severe asthma exacerbation also requires hospitalization of the patient or at least an emergency room visit.

Participants/ population

Participants will include individuals of both sexes and all ages exposed to different pollution levels. Persons with known concomitant conditions that may interfere in the association between asthma and air pollution will be excluded.

Intervention(s), exposure(s)

The exposure will be traffic-related or industrial air pollution, measured as an increase in the air concentration of the most frequently monitored air pollutants: nitrogen dioxide (NO2), sulfur dioxide (SO2), carbon monoxide (CO), ozone (O3) and suspended particulate matter (PM10 and PM2.5).

Comparator(s)/ control

Not applicable.

Context

Giving the high risk of bias potentially associated with observational study designs, only the articles published in indexed peer-reviewed journals will be included. Additionally, a careful assessment of the study quality will be performed, in order to ensure the validity of the results.

Outcome(s)

Primary outcomes

Severe asthma exacerbations are defined as an episode of wheezing or shortness of breath that include the need for oral or systemic corticosteroids, and require hospitalization or an emergency room visit.

Secondary outcomes

None.

Data extraction, (selection and coding)

Two independent reviewers (PO and JR) will screen the retrieved articles by title and abstract to complete the first stage, and select those articles that potentially meet the inclusion criteria. In a second stage the same reviewers will read the full text of the primary selected articles to evaluate the inclusion of data for meta-analysis. Any disagreement will be discussed and resolved by consensus or arbitration by a third investigator (BB). Reasons for exclusion will be recorded for each article. In case of missing data, the corresponding author of the study will be contacted.

A data extraction form will be developed in Excel, and filled in with information of each study. This information will include: first author, year of publication, country, participant characteristics, outcome measures and exposure. Other variables that will be included are the lag time between the exposure and the outcome, latitude, elevation, annual concentration of main pollutants and other variables that might be acting as moderators.

Risk of bias (quality) assessment

The quality of included studies will be assessed by two independent reviewers (PO and JR) using a modified Newcastle-Ottawa Scale for case-control studies in meta-analysis, with a scale ranging from 1 to 9 stars (1–3 stars for poor quality, 4-6 for intermediate and 7-9 for high quality). Any disagreement will be discussed and resolved by consensus or arbitration by a third investigator (BB).

The risk of publication bias and other biases will be assessed checking the symmetry of funnel plots for each pollutant, and through regression methods like the Egger's test and the Rank correlation test. The impact of missing studies in the association measure will be assessed and corrected using the trim and fill method.

Methods for removing outlier studies based on regression diagnostic techniques adapted for meta-analysis will be followed. These will include the Cook's distance and the analysis of regression residuals. Outlying studies will be excluded from the meta-analysis.

Strategy for data synthesis

The retrieved odds ratio for each study will be normalized to represent the difference in each pollutant's concentration at a same scale between case and control periods. The natural logarithm and standard errors of the odds ratios will be calculated based on the 95% confidence intervals mentioned in each article. A summary measure of association will be estimated considering three models: a random effect model, a mixed model using co-variables as moderators (meta-regression analysis), and a multilevel mixed model that will also take into account the inclusion of odds ratios from different subgroups within a same study. The mixed model and the multilevel mixed model will include the time lag between the exposure and the outcome, the latitude and elevation of the city where the study was performed, the annual outdoor concentration of each selected pollutant, and the year of publication as potential moderators that could explain the heterogeneity between the studies. The best nested model will be selected using the likelihood ratio test. The heterogeneity between the studies will be analyzed with the statistic I-squared, considering moderate heterogeneity with values > 30% and severe heterogeneity with values > 50%.

A sensitivity analysis will be carried out by means of repeatedly fit of the random effect model, leaving out one study at a time and assessing the impact of this procedure on the summary measure. If possible, a sensitivity analysis based on the quality of included studies will also be performed.

Analysis of subgroups or subsets
None planned.

Dissemination plans
A manuscript with the study results will be submitted to a specialized peer-reviewed journal in this field.

Contact details for further information
Dr Orellano

Universidad Tecnologica Nacional, Facultad Regional San Nicolas, Colon 332, San Nicolas, Provincia de Buenos Aires, Argentina (B2900LWH).

porellano@gmail.com

Organisational affiliation of the review
Universidad Tecnologica Nacional, Facultad Regional San Nicolas and Consejo Nacional de Investigaciones Cientificas y Tecnicas, Argentina.

http://www.frsn.utn.edu.ar/frsn/index.asp

Review team
Dr Pablo Orellano, Universidad Tecnologica Nacional and Consejo Nacional de Investigaciones Cientificas y Tecnicas, San Nicolas, Argentina.
Dr Julieta Reynoso, Hospital Interzonal General de Agudos "San Felipe", San Nicolas, Argentina.
Dr Brenda Balbi, Hospital Interzonal General de Agudos "San Felipe", San Nicolas, Argentina.
Dr Julia Vasquez, Hospital Interzonal General de Agudos "San Felipe", San Nicolas, Argentina.
Dr Nancy Quaranta, Universidad Tecnologica Nacional and Comision de Investigaciones Cientificas de la Provincia de Buenos Aires, San Nicolas, Argentina.

Anticipated or actual start date
02 November 2015

Anticipated completion date
30 June 2016

Funding sources/sponsors
Consejo Nacional de Investigaciones Cientificas y Tecnicas (CONICET), Argentina.

Conflicts of interest
None known

Other registration details
This study will be registered in the Argentina’s national registry for health research (Registro Nacional de Investigaciones en Salud).

Language
English

Country
Argentina

Subject index terms status
Subject indexing assigned by CRD

Subject index terms
Air Pollutants; Air Pollution; Asthma; Cross-Over Studies; Humans

Stage of review
Ongoing

Date of registration in PROSPERO
16 December 2015

Date of publication of this revision
16 December 2015

Stage of review at time of this submission	Started	Completed
Preliminary searches	Yes	Yes
Piloting of the study selection process	Yes	Yes
Formal screening of search results against eligibility criteria	No	No
Data extraction	No	No
Risk of bias (quality) assessment	No	No
Data analysis	No	No

PROSPERO
International prospective register of systematic reviews
The information in this record has been provided by the named contact for this review. CRD has accepted this information in good faith and registered the review in PROSPERO. CRD bears no responsibility or liability for the content of this registration record, any associated files or external websites.
