# Supplementary material for: Effect of outdoor air pollution on asthma exacerbations in children and adults: Systematic review and multilevel meta-analysis
Source: PLoS One. 2017 Mar 20;12(3):e0174050. doi: 10.1371/journal.pone.0174050 (PMC5358780; doi:10.1371/journal.pone.0174050)
Supplement: S2 File — (PDF) [file pone.0174050.s002.pdf]

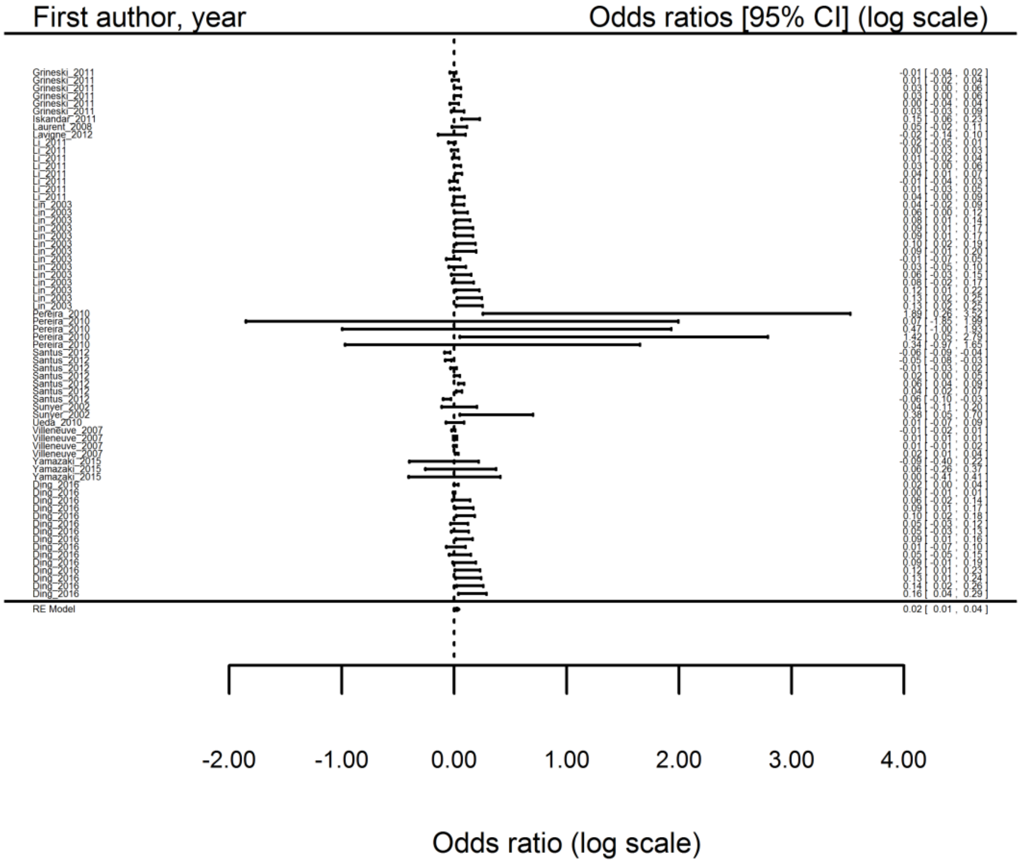

Forest plot of 13 studies examining the association between NO_2 and asthma exacerbations.

Each study reports one or more time-lags. The pooled odds ratio in logarithmic scale is indicated at the bottom of the figure.

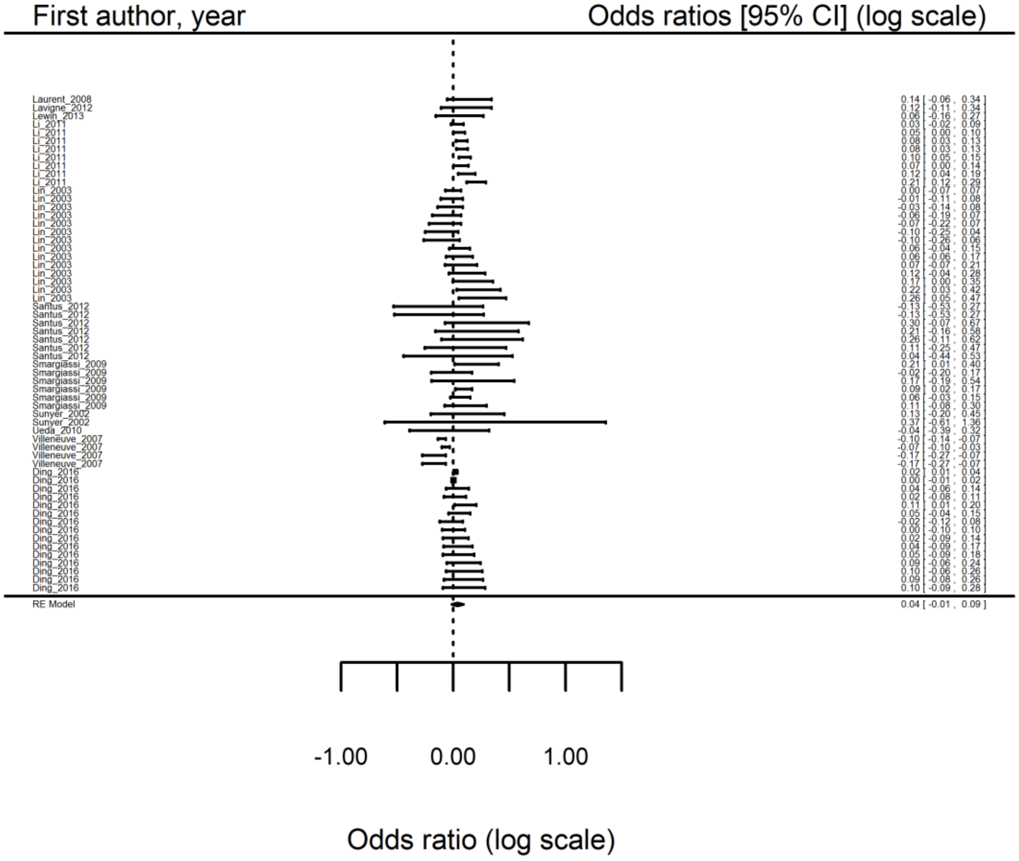

Forest plot of 11 studies examining the association between SO_2 and asthma exacerbations.

Each study reports one or more time-lags. The pooled odds ratio in logarithmic scale is indicated at the bottom of the figure.

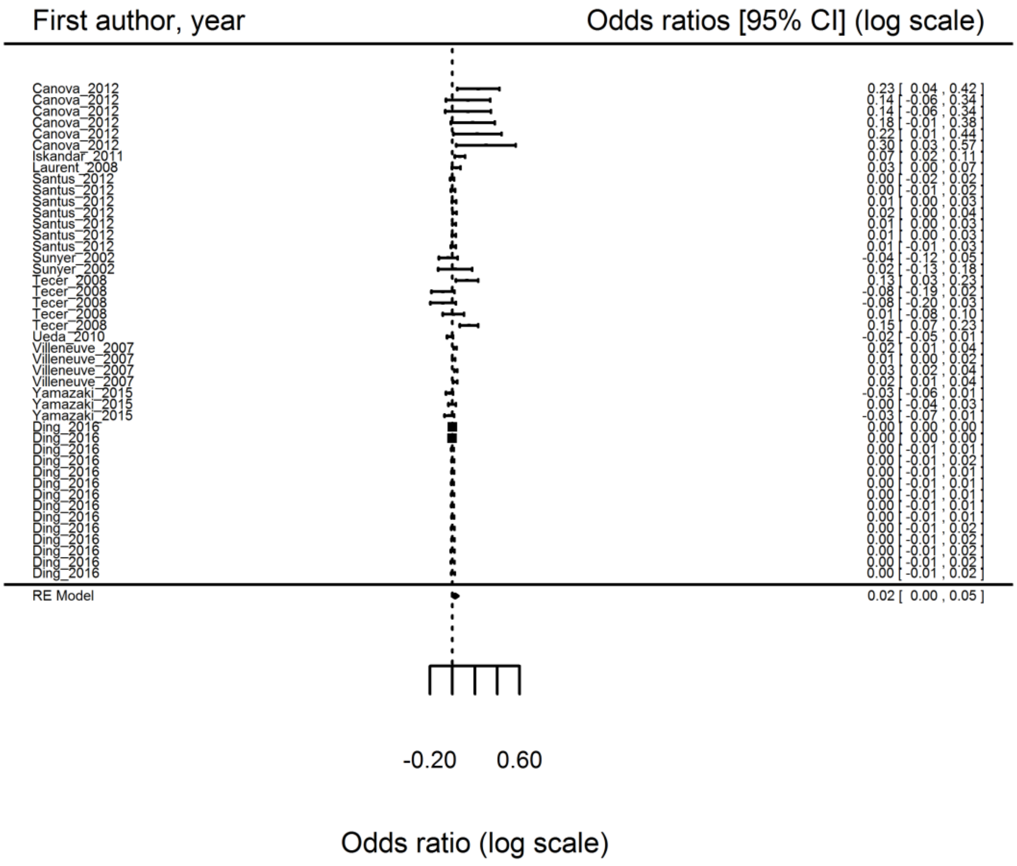

Forest plot of 10 studies examining the association between PM_{10} and asthma exacerbations.

Each study reports one or more time-lags. The pooled odds ratio in logarithmic scale is indicated at the bottom of the figure.

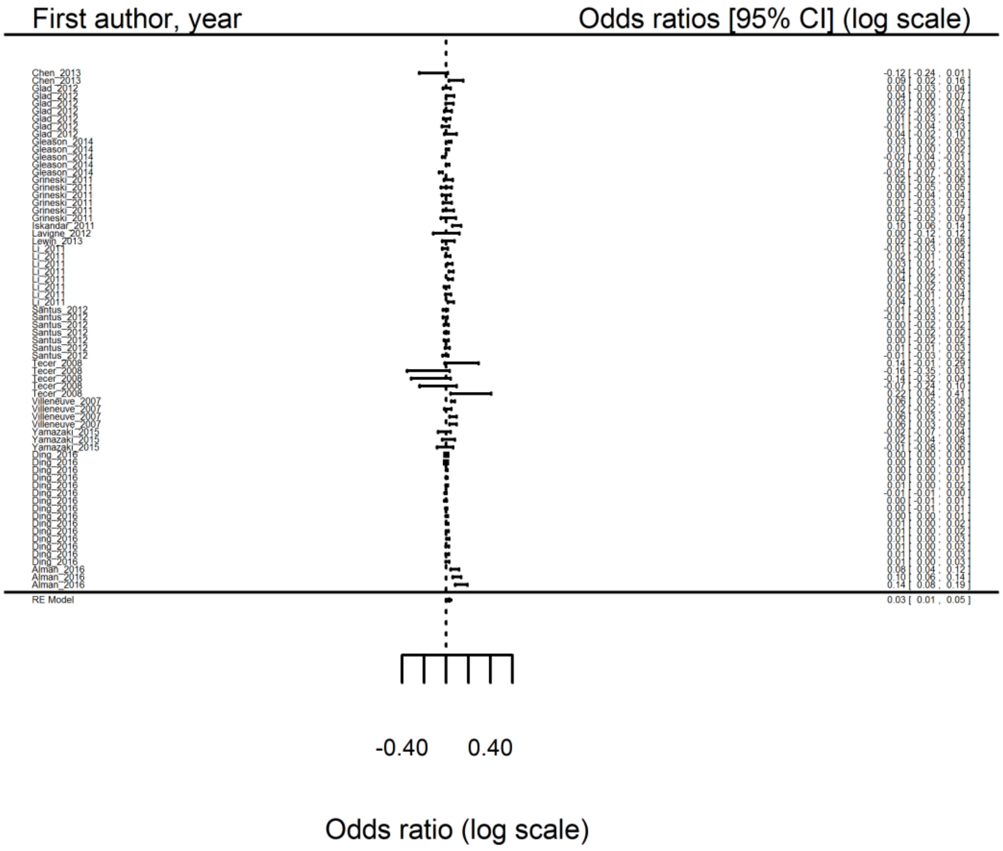

Forest plot of 14 studies examining the association between $PM_{2.5}$ and asthma exacerbations.

Each study reports one or more time-lags. The pooled odds ratio in logarithmic scale is indicated at the bottom of the figure.

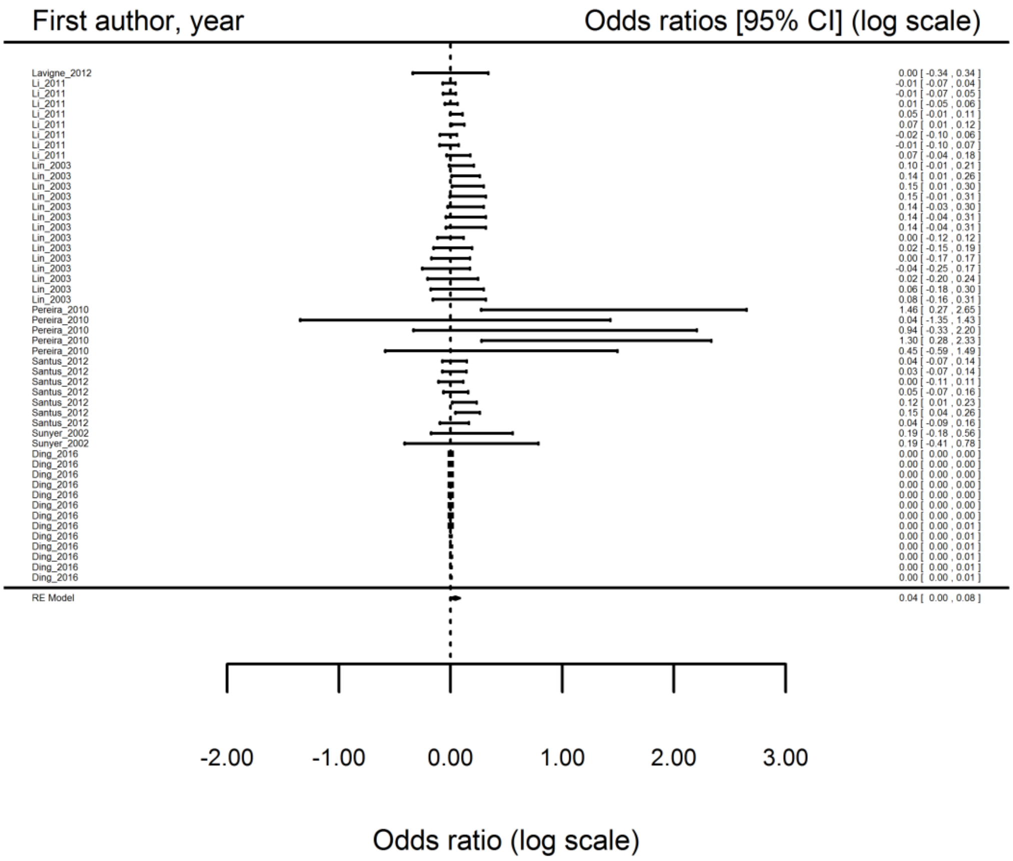

Forest plot of 7 studies examining the association between CO and asthma exacerbations.

Each study reports one or more time-lags. The pooled odds ratio in logarithmic scale is indicated at the bottom of the figure.

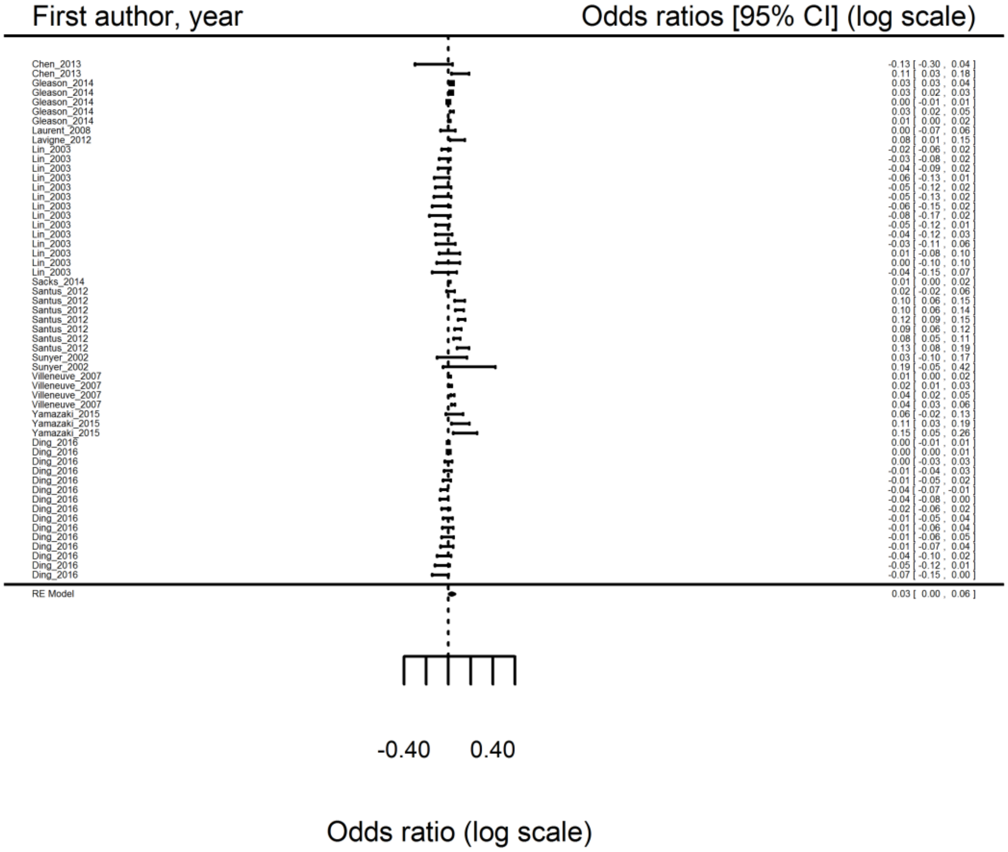

Forest plot of 11 studies examining the association between O_3 and asthma exacerbations.

Each study reports one or more time-lags. The pooled odds ratio in logarithmic scale is indicated at the bottom of the figure.
