# Supplementary material for: Effect of outdoor air pollution on asthma exacerbations in children and adults: Systematic review and multilevel meta-analysis
Source: PLoS One. 2017 Mar 20;12(3):e0174050. doi: 10.1371/journal.pone.0174050 (PMC5358780; doi:10.1371/journal.pone.0174050)
Supplement: S2 Table — (PDF) [file pone.0174050.s004.pdf]

S2 Table. Detailed literature search.

October 17, 2016

**PubMed**<br><br>(asthma [Title/Abstract] OR wheeze[Title/Abstract]) AND (pollut*[Title/Abstract] OR contamin*[Title/Abstract]) AND (hospitaliz*[Title/Abstract] OR admission*[Title/Abstract] OR emergenc*[Title/Abstract] OR attack*[Title/Abstract]) AND "case crossover" [Title/Abstract]
**Scopus**<br><br>TITLE-ABS-KEY ((asthma OR wheeze) AND (pollut* OR contamin*) AND (hospitaliz* OR admission* OR emergenc* OR attack*) AND "case crossover")
**Google Scholar**<br><br>(intitle:asthma OR intitle:wheeze) AND (intitle:pollutants OR intitle:pollution OR intitle:contaminants OR intitle:contamination) AND (intitle:hospitalizations OR intitle:admissions OR intitle:emergency OR intitle:attack) AND ("case crossover")
