# Supplementary material for: Effect of outdoor air pollution on asthma exacerbations in children and adults: Systematic review and multilevel meta-analysis
Source: PLoS One. 2017 Mar 20;12(3):e0174050. doi: 10.1371/journal.pone.0174050 (PMC5358780; doi:10.1371/journal.pone.0174050)
Supplement: S3 Table — (PDF) [file pone.0174050.s005.pdf]

S3 Table. Newcastle-Ottawa criteria.

NOS question	NOS criteria applied to this study
Selection	
Is the case definition adequate?	Revision of clinical histories or primary record sources (1 star)
Representativeness of the Cases	Cases from all hospitals or clinics, or a representative sample of cases (1 star)
Selection of Controls	Always 1 star
Definition of Controls	Exclusion of patients with more than one exacerbation during a given period of time (1 star)
Comparability	
Comparability of cases and controls on the basis of the design or analysis	Always 1 star<br><br>Control by the day of the week and temperature or humidity (1 additional star)
Exposure	
Ascertainment of exposure	Residential address of cases was verified (1 star)
Same method of ascertainment for cases and controls	Always 1 star
Non-response rate	Always 1 star

NOS: Newcastle-Ottawa scale.
