# Supplementary material for: Effect of outdoor air pollution on asthma exacerbations in children and adults: Systematic review and multilevel meta-analysis
Source: PLoS One. 2017 Mar 20;12(3):e0174050. doi: 10.1371/journal.pone.0174050 (PMC5358780; doi:10.1371/journal.pone.0174050)
Supplement: S4 Table — (PDF) [file pone.0174050.s006.pdf]

S4 Table. Multilevel meta-regression for the sensitivity analysis.

Pollutant	Moderators	Q_E (P-value)
NO_2	Lag + Latitude + Elevation	0.33
SO_2	Lag + Latitude + Elevation	0.16
PM_{10}	Lag + Latitude + Elevation	0.68
$PM_{2.5}$	Lag + Latitude + Elevation	0.06
CO	Lag + Latitude + Elevation	0.99
O_3	Lag + Latitude + Elevation	0.12

NO_2: nitrogen dioxide; SO_2: sulfur dioxide; O_3: ozone; CO: carbon monoxide; PM_{10}: particulate matter < 10 µm; $PM_{2.5}$: particulate matter < 2.5 µm; Q_E: test for residual heterogeneity.
